# Study of Synthesis of Dual-Curing Thermoplastic Polyurethane Hot-Melt Adhesive and Optimization by Using Gray Relational Analysis to Apply in Fabric Industry to Solve Seamless Bonding Issues

**DOI:** 10.3390/polym16040467

**Published:** 2024-02-07

**Authors:** Sheng-Yu Lin, Naveed Ahmad, Chung-Feng Jeffrey Kuo

**Affiliations:** Department of Materials Science and Engineering, National Taiwan University of Science and Technology, Taipei 10607, Taiwan; m10804206@mail.ntust.edu.tw (S.-Y.L.); bajwanaveed145@gmail.com (N.A.)

**Keywords:** gray relational analysis, thermosetting polyurethane, Taguchi quality engineering method, polyhydric alcohols, dual-cure photothermosetting polyurethane, signal-to-noise ratio

## Abstract

People wear clothes for warmth, survival and necessity in modern life, but in the modern era, eco-friendliness, shortened production times, design and intelligence also matter. To determine the relationship between data series and verify the proximity of each data series, a gray relational analysis, or GRA, is applied to textiles, where seamless bonding technology enhances the bond between components. In this study, a polyurethane prepolymer, 2-hydroxyethyl acrylate (2-HEA) as an end-capping agent and n-octyl acrylate (ODA) as a photoinitiator were used to synthesize a dual-curing polyurethane hot-melt adhesive. Taguchi quality engineering and a gray relational analysis were used to discuss the influence of different mole ratios of NCO:OH and the effect of the molar ratio of the addition of octyl decyl acrylate on the mechanical strength. The Fourier transform infrared spectroscopy (FTIR) results showed the termination of the prepolymer’s polymerization reaction and the C=O peak intensity at 1730 cm^−1^, indicating efficient bonding to the main chain. Advanced Polymer Chromatography (APC) was used to investigate the high-molecular-weight (20,000–30,000) polyurethane polymer bonded with octyl decyl acrylate to achieve a photothermosetting effect. The thermogravimetric analysis (TGA) results showed that the thermal decomposition temperature of the polyurethane hot-melt adhesive also increased, and they showed the highest pyrolysis temperature (349.89 °C) for the polyhydric alcohols. Furthermore, high peel strength (1.68 kg/cm) and shear strength (34.94 kg/cm^2^) values were detected with the dual-cure photothermosetting polyurethane hot-melt adhesive. The signal-to-noise ratio was also used to generate the gray relational degree. It was observed that the best parameter ratio of NCO:OH was 4:1 with five moles of monomer. The Taguchi quality engineering method was used to find the parameters of single-quality optimization, and then the gray relation calculation was used to obtain the parameter combination of multi-quality optimization for thermosetting the polyurethane hot-melt adhesive. The study aims to meet the requirements of seamless bonding in textile factories and optimize experimental parameter design by setting target values that can effectively increase production speed and reduce processing time and costs as well.

## 1. Introduction

Adhesives are used in the production of thousands of textile items that we employ daily, including conveyor belts, sportswear, casual wear, protective clothes and swimwear. Textiles and garments have become necessities in the modern world. People used to wear clothing to remain warm and to survive. However, as modern technology has advanced, consumers increasingly desire comfort when wearing clothes in addition to design, eco-friendliness, functionality, convenience and intelligence. Manufacturers seek to have more extensive design options, lower labor costs and shorter manufacturing timeframes. The majority of traditional textile processing techniques stitch together materials employing the sewing method with needles and threads. Wrinkles, skipped stitches, uneven seams, broken threads and other flaws could appear during processing. It can even be detrimental to wear some useful clothes together [1]. Therefore, the use of seamless bonding technology in textiles can not only strengthen the bonds between fabrics but also shorten the processing time and avoid gaps. The use of adhesive technology involves using adhesive materials to quickly and tightly bond two fabrics together, which can replace the seams and fasteners commonly used in clothing. It can lessen the discomfort for the average consumer by minimizing friction between skin and seams, and it can provide designers and manufacturers with more alternatives regarding how clothing looks. As a result, a seam-free processing technique using adhesives commonly utilized in a variety of sectors has arisen in recent years. Adhesives have many advantages over traditional methods, including fatigue resistance, design flexibility and environmental endurance. Adhesives are therefore used in many different industries, such as building, oil, sports, electronics, aviation, cars and clothing adhesion [2,3,4].

Currently, there are still some challenges in the industry regarding seamless bonding technology. This study summarizes the common defects in seamless bonding provided by textile factories into the following four points: (i) The hot-melt adhesive cannot bond tightly with the substrate, resulting in insufficient adhesion and curling. (ii) During the process of melt pressing and lamination, the base material may produce visible defects. (iii) After the process of melt and pressure lamination, the fabric may shrink and have a poor appearance. (iv) The melting temperature of the hot-melt adhesive is too high, resulting in severe color differences in the fabric. Based on the above issues, it is clear that the defects are all caused by the high melting temperature of the hot-melt adhesive. Therefore, softening the processing temperature of the hot-melt adhesive will be one of the important breakthroughs in seamless bonding technology.

Polyurethane has extensive applications in several industries, such as medicine, maritime, plumbing systems, footwear, rigid as well as flexible foam, sealants, adhesives, insulators and many more [5,6,7,8]. Adhesives for flooring, roofing, doors and windows, shoes, foams, coatings, elastomers, soft packaging, glass, textiles, etc., typically contain polyurethane [9,10,11,12]. Polyurethane, formed by a polymerization reaction, contains a large number of urethane bonds. Urethane bonds are formed by the reaction between the -NCO group of isocyanates and the hydroxyl group of polyols [13,14,15]. The structure of thermoplastic polyurethane comprises a linear copolymer composed of alternating flexible soft-chain segments and rigid hard-chain segments. The soft-chain segments are composed of long-chain polyols, which can be polyethers such as polyethylene glycol, polypropylene glycol, polytetrahydrofuran and polyester polyols [3]. The hard-chain segments are composed of polyisocyanates and small-molecule alcohols. Due to the thermodynamic incompatibility between the two types of chain segments during polymerization, a micro-phase separation structure is formed, which makes thermoplastic polyurethane have unique mechanical properties, high durability, excellent chemical resistance and easy processing and application [16]. The most common method of seamless processing for textiles is the use of adhesive hot-melt film/strips. A hot-melt adhesive is a 100% solid thermoplastic material that can become sticky when heated. The process involves heating and melting the adhesive, which is then applied to the substrate to be bonded. The adhesive adheres to the substrate, and the two materials are bonded together, and then it cools and solidifies to complete the bonding process. It was found in their study that the hydroxyl group of a polyurethane hot-melt pressure-sensitive adhesive can form a chemical bond with the nylon fiber substrate, which has polar functional groups, further improving the adhesion between the adhesive and the substrate. It also exhibited strong adhesion in terms of peel strength and shear strength.

When wearing tight-fitting clothes, friction between the body and the seams may cause discomfort. Seamless bonding technology can effectively decrease these sewing areas’ vulnerabilities in feasible clothing [17]. Compared with traditional hot-melt adhesives, polyurethane hot-melt adhesives are block or multi-block copolymers formed by alternating flexible and rigid chain segments. Their characteristic lies in their structure, which contains a high content of urethane and urethane ester bonds. Polyurethane hot-melt adhesives have many advantages, such as being environmentally friendly; easy to use and process; effectively wetting the surface of many substrates; diffusing through porous substrates; having good toughness, water resistance, impact resistance and chemical resistance; and forming covalent bonds with substrates containing active hydrogen [7,18].

This study is focused on raw materials and the curing methods of polyurethane hot-melt adhesives to overcome different problems. Polyurethane hot-melt adhesives were synthesized by using three different types of polyols. 2-HEA and ODA form hydrogen bonding interactions through C=C groups with the functional groups on nylon fiber, which can enhance the adhesion performance as well as the microphase separation degree, which ultimately strengthens the adhesive. In addition to this, the alkyl chain group in the photoinitiator ODA is used to enhance the resistance of water to adhesives. Different techniques, i.e., FTIR, APC, TGA and moisture content determination, were used to analyze the results. By defining the desired objectives for significant characteristics, the study intends to meet the requirements of seamless bonding in textile manufacturing. The NCO/OH ratio, three different categories of polyols and the amount of photocurable monomer added are selected as control factors. According to studies by Gogoi et al. [19] and Somani et al. [20], the NCO/OH ratio is mostly between 3:1 and 1:1. The three types of polyols used for the soft segment of this study are traditional polyester, polyether and a newer polycarbonate. The aim is to determine which type of polyol provides excellent adhesion to nylon fabric. n-octyl acrylate (ODA) is used to achieve crosslinking through the C=C groups shared by 2-HEA and ODA under UV light stimulation. Additionally, the longer alkyl chain of ODA can increase the water resistance of the adhesive and improve its wetting properties on substrates. Lastly, the gray relational analysis method was used to create a multi-quality optimization parameter design for the dual-cure polyurethane hot-melt adhesive to optimize its mechanical properties, such as its peel strength and shear strength.

## 2. Materials and Methods

### 2.1. Materials

Polytetramethylene glycol (PTMG, Mitsubishi Chemicals Group, Tokyo, Japan), polycaprolactone (PCL Sigma-Aldrich, St. Louis, MO, USA) and poly (hexamethylene carbonate) diol (PHCD, Henan Tianfu Chemicals Co., Ltd., Zhengzhou, China) were used as the soft-chain segment monomers. Diphenylmethane diisocyanate (MDI, Hubei Pretty Chemicals Technology, Co., Ltd., Wuhan, China) was used as the rigid-chain segment monomer, and 2-hydroxyethyl acrylate (2-HEA, Tokyo Chemical Industry Co., Ltd., Tokyo, Japan) was used as the end-capping agent for n-octyl acrylate (ODA, Osaka Organic Chemical Industry, Ltd., Osaka, Japan), with di-ionized water.

### 2.2. Methods

In the quality characteristic optimization, the NCO/OH ratio, three different categories of polyols and the amount of photocurable monomer added are selected as control factors. The levels of each control factor are selected according to the literature. The synthesis parameter setting ranges are shown in Table 1 and Table 2, and the Taguchi L9 orthogonal array used for the experimental design is shown in Table 3. In this study, the levels of the NCO/OH ratio were set at 2:1, 3:1 and 4:1. The three types of polyols used in the soft segment of this study are traditional polyester, polyether and the newer polycarbonate. The concentration levels of n-octyl acrylate (ODA) were set at 0.1, 0.5 and 0.15 moles.

#### 2.2.1. Synthesis Reaction of Polyurethane Hot-Melt Adhesives

**Figure 1:**
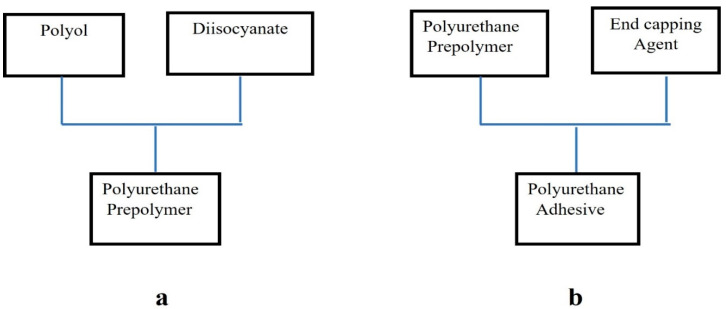
Synthesis of polyurethane hot-melt adhesive; (**a**) combination of a diisocyanate and polyol; (**b**) combination of a polyurethane prepolymer and End-capping Agent.

Polyurethane hot-melt adhesives are synthesized by the prepolymer method, as shown in Figure 1, which has two steps (a, b). Step (a) involves a reaction between a diisocyanate and polyol to form a polyurethane prepolymer, a low-melting and low-molecular-weight adhesive substance. In Step (b), the polyurethane prepolymer further reacts with an end-capping agent to produce a high-melting and high-molecular-weight polyurethane polymer. A polyurethane hot-melt adhesive synthesized by this method has better comprehensive properties. During this preparation process, three polyols, i.e., polytetramethylene glycol (PTMG), polycaprolactone (PCL) and poly(hexamethylene carbonate)diol (PHCD), are used as the soft-chain segment monomers. Diphenylmethane diisocyanate (MDI) was used as the rigid-chain segment monomer, and 2-hydroxyethyl acrylate (2-HEA) was used as an end-capping agent to terminate the polyurethane prepolymer reaction. Finally, a suitable amount of n-octyl acrylate (ODA) was added as a photocurable monomer to synthesize a series of dual-cure polyurethane hot-melt adhesives.

#### 2.2.2. Methodology

**Figure 2:**
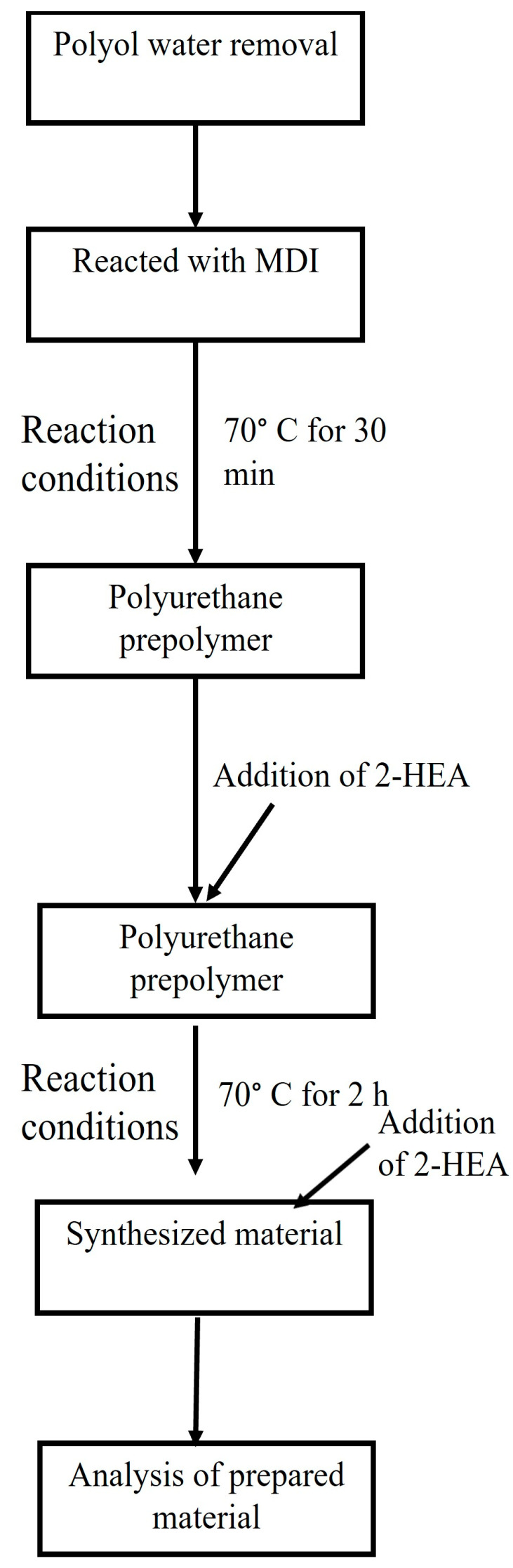
Experimental flowchart of cured dual-curing polyurethane hot-melt adhesive.

In this study, vacuum drying was used to remove water from the polytetrahydrofuran polyol, polycaprolactone diol, polycarbonate diol, isocyanate, hydroxyethyl methacrylate and octyl decyl acrylate. The monomers were placed in a vacuum distillation apparatus and subjected to vacuum drying for 0.5 to 14 h at 100 °C. The water content was calculated, and a synthesis was conducted when the water content was below 0.05%. A flowsheet of the methodology is explained in Figure 2.

**Figure 3:**
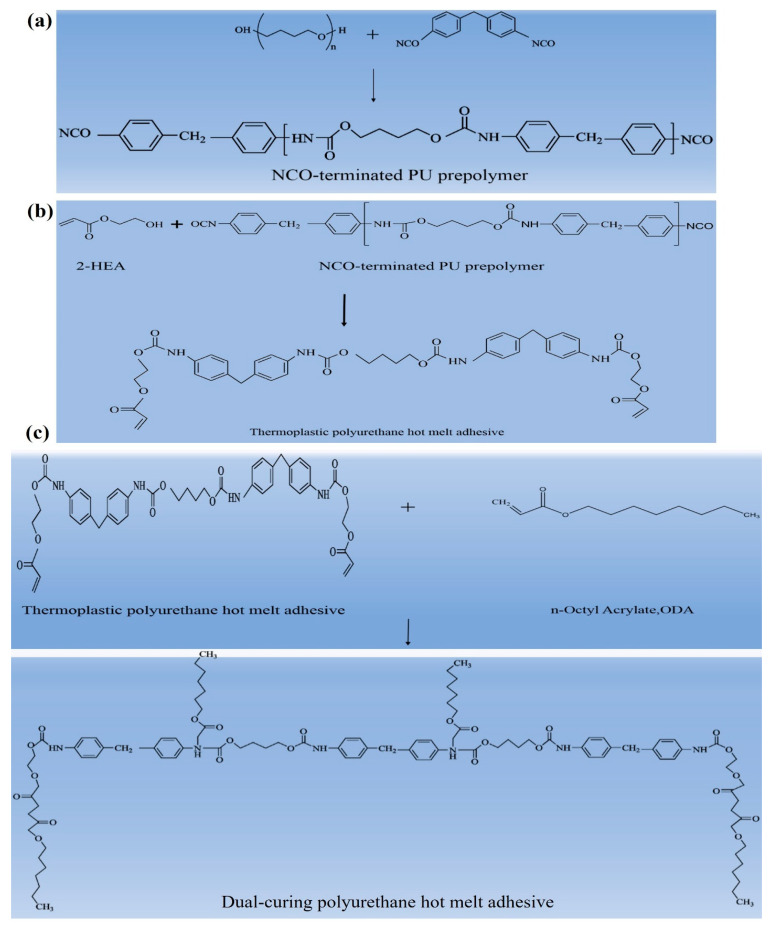
Chemical reaction of synthesis of polyurethane hot-melt adhesive. (**a**) Reaction synthesis of NCO-terminated PU prepolymer. (**b**) Reaction synthesis of thermoplastic polyurethane hot-melt adhesive. (**c**) Reaction synthesis of dual-cure polyurethane hot-melt adhesive.

In the first step, the reaction flask was placed in a 75 °C oil bath and vacuum-degassed completely before being filled with nitrogen gas. Then, excess molten MDI and dehydrated polyol were added to the reaction flask in sequence and mixed thoroughly using a stirring motor at a speed of 500 rpm for 30 min. Subsequently, ethyl acetate was added as a solvent to adjust the viscosity for 10 min to obtain the polyurethane prepolymer. The NCO content in the prepolymer was determined by the standard dibutylamine back-titration method. The second step involved maintaining the temperature at 75 °C and introducing nitrogen to maintain an inert environment. The prepolymer and hydroxyethyl methacrylate were added and stirred continuously at 500 rpm for 2 h. Finally, an appropriate amount of a photopolymerizable monomer, octyldecyl acrylate, was added and stirred for 30 min. Vacuum defoaming was then carried out, and the dual-curing polyurethane hot-melt adhesive was poured into a PTFE mold. After being kept at room temperature for three days, the fully cured dual-curing polyurethane hot-melt adhesive could be obtained and was subjected to a chemical property-related analysis. The main steps of the polymerization method were used to carry out a two-step core polymerization method. The chemical reaction of polymerization is given in Figure 3a–c.

### 2.3. Polyhydric Alcohol Dehydration Time

To avoid explosive polymerization and unstable aminoformate group decomposition, vacuum drying was used to remove water from the polyols, which were placed in a vacuum concentrator at 100 °C and remained there for 0.5–14.0 h. The water content was measured by using the following equation.

(1) $$\mathrm{Moisture}\;(\%)=\frac{W_{damp\;-\;W_{dry}}}{W_{damp}}\times 100\%$$

where W_damp_ is the weight before drying and W_dry_ is the weight after drying.

**Figure 4:**
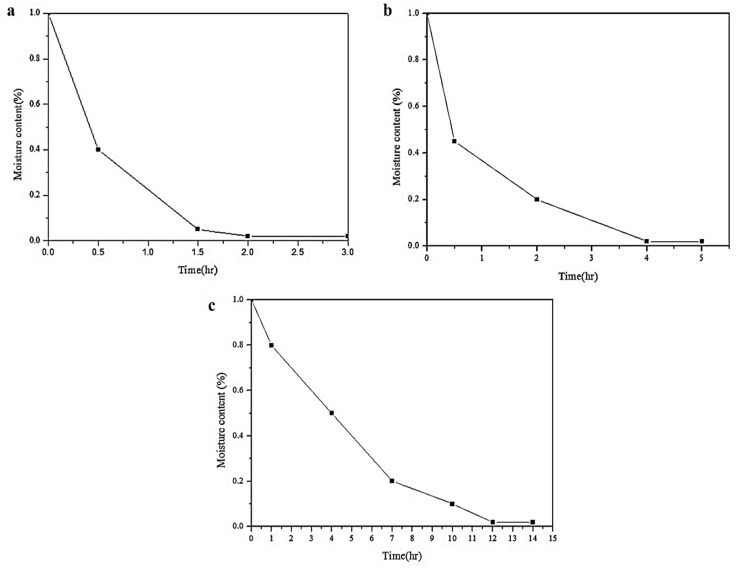
Graphs for Moisture content of (**a**) PTMG, (**b**) PCL and (**c**) PHCD at different drying times (it shows the effects of time on the content of water).

Figure 4 shows the variation curves of the water removal time for the three types of soft-chain segment polyols. As seen from Figure 4a, the water content of PTMG drops significantly in just 1.5 h and becomes stable after 2 h. On the other hand, as shown in Figure 4b, PCL requires 4 h to stabilize. Finally, due to its highest viscosity, PHCD requires 12 h to become stable and no longer vary, as shown in Figure 4c. To ensure experimental stability, in this study, an additional hour was added to the water removal time for each polyol to ensure stability before conducting experiments.

### 2.4. Taguchi Quality Engineering Method

The core concept of the Taguchi engineering method is to design experiments by setting experimental parameters (control factors and their levels) using an orthogonal table, calculating the signal-to-noise ratio (*S*/*N* ratio) and using a main effect analysis and variance analysis to consider the impact of both the mean values and variances, properly handling noise factors, obtaining the optimal combination of control factors and achieving the goal of saving experimental time and cost while obtaining important parameters that affect the experimental results. The Taguchi quality engineering method greatly reduces the number of experiments needed using orthogonal arrays while also reducing the variability of the process and quality factors, resulting in stable and cost-effective testing results. The Taguchi experimental design methodology operates via orthogonal array design and parameter design. The control factors, signal factors and noise factors are part of the parameter design. The most common representation of the Taguchi orthogonal table is La(b^c^), where L is the first letter of Latin squares, a is the number of experiments required after the statistical analysis, b is the number of levels of the controlled factors in the experiment and c is the maximum number of factors that can be calculated. A basic orthogonal table of the Taguchi method and Taguchi orthogonal array is shown in Tables S1 and S2 (Supporting Document).

There are a number of factors, i.e., control factors, signal factors, noise factors and interference factors, for the Taguchi quality engineering technique, but the quality loss function is also important, which includes smaller-the-better (STB), larger-the-better (LTB) and nominal-the-best (NTB) types [21]. STB means the quality characteristics are smaller and the quality will be better, which includes the hot-melt adhesive and the amount of overflow. The LTB characteristics explain that when the quality characteristics are larger, the quality is better, which includes the shear strength and peel strength of the adhesives. The quality loss function for the NTB characteristics includes the softening point of adhesives and the ideal target value (m). As a quality characteristic begins to deviate from its target value, the quality loss function also increases quadratically, as shown in the equations below. The STB, LTB and NTB graphs are shown in Figure S1a–c (Supplementary Document) and are represented as below

L(s) = ks^2^ (2)

(3) $$L(s)=k\frac{1}{s^{2}}$$

L(s) = k(s − m)^2^ (4)

When the quality loss function approaches the target value, the product’s quality loss decreases.

### 2.5. Physical Analysis

According to the ASTM D36 standard [22], a softening point test was performed by placing the sample in a metal ring-shaped container (diameter above 15.9 mm; thickness of 2.38 mm; depth of 6.35 mm), and a steel ball weighing 3.5 g was placed above the sample’s center. The container was then heated at a rate of 5 °C per minute, and the temperature at which the steel ball dropped was recorded as the softening point. The softening point temperature is affected by the heating rate. The peel strength test is conducted according to the ASTM D3330 standard [23] using a universal testing machine (a tensile tester) with an Orientec RTA-1T model. The test mainly measures the different tensile properties of polymers. A polyurethane hot-melt adhesive strip is cut to a size of 1 cm × 15 cm, and a nylon cloth is cut to a size of 2.54 cm × 18 cm. The following processing conditions are used for bonding: First, after the nylon cloth and adhesive strip are fixed, they are placed under a UV machine for precise positioning and the first polymerization cross-linking. The exposure time is 2 s. Then, the product is placed on a heat press at a processing temperature of 100 °C for a processing time of 20 s. with a processing pressure of 1 kg/cm^2^. After bonding, it is allowed to stand for 20 min to cool and stabilize, and then the peel strength is tested at a speed of 300 mm/min. According to the specification CNS 8306 L3141, the shear strength test is performed using a universal tensile testing machine (Orientec RTA-1T). Polyurethane hot-melt adhesive tape is cut into 1 cm × 15 cm, and nylon fabric is cut into 2.54 cm × 18 cm. The following processing conditions are applied: after fixing the nylon fabric and adhesive tape, they are placed under the UV machine for precise positioning and the first polymerization and crosslinking process, with a UV exposure time of 2 s. Then, they are placed on a hot press at a processing temperature of 100 °C for a processing time of 20 s with a processing pressure of 1 kg/cm^2^. After bonding, it is left to stand for 20 min to cool and stabilize, and a shear strength test is performed at a speed of 300 mm/min. Same procedure is repeated to check the peel strength (Section S1.1 Signal-to-Noise Ratio, Section S1.2 Main Effects Analysis, Section S1.3 Analysis of Variance, Section S1.4 Calculation of Grey Relational Analysis and Section S1.5 Industry Requirements; theoretical and statistical explanations and formulas discussed in the Supplementary Materials).

### 2.6. Gray Relational Analysis (GRA)

The present study aims to optimize the parameter design for the polymerization reaction of dual-cured polyurethane hot-melt adhesive and focuses on the quality characteristics of shear strength and peel strength when applied to nylon fabric.

#### 2.6.1. Gray Relational Analysis (GRA) Method

The Taguchi quality method is used to configure the experimental parameters for the polymerization reaction of the dual-cured polyurethane hot-melt adhesive. The Taguchi engineering method’s L_9_ orthogonal table is used to adjust the experimental parameters. A GRA is able to calculate the degree of correlation between data series with a small amount of data and use the differences between factors to calculate the correlation between data series, making up for the shortcomings of traditional analysis methods. Before conducting the gray relational analysis, the sequences must satisfy comparability to perform the analysis. Assuming that Xi and Xj represent two comparable gray relational degree sequences to be calculated, γ (Xi, Xj) is used to represent the gray relational degree between Xi and Xj. There are four necessary conditions for a gray correlation analysis, namely consistency, symmetry, comprehensiveness and closeness, with their formulae as follows:

(5) Consistency: 0 < γ (Xi, Xj) ≤ 1

When γ (Xi, Xj) = 0 in the gray correlation system, it means that there is no correlation between the two sequences Xi and Xj; when γ (Xi, Xj) = 1, it means that the two sequences are completely correlated.

Symmetry: γ (Xi, Xj) = γ (Xj, Xi)(6)

When the gray correlation system has three or more sequences, because the selected sequences may be different, the comprehensiveness usually satisfies the formula:

(7) Comprehensiveness: γ (Xi, Xj) ≠ γ (Xj, Xi)

Closeness: The smaller the absolute value of Xi − Xj, the larger the value of γ (Xi, Xj). In this case, γ (Xi, Xj) can be referred to as the gray correlation coefficient between sequence Xj and sequence Xi under the conditions.

#### 2.6.2. Optimization Quality Analysis Process

There are a total of nine experimental groups, with three levels of water content, isocyanate-to-polyol ratios and photoinitiator additive amounts. The three levels are for polyether, polyester and polycarbonate as the soft-chain segment polyols, and the NCO:OH, ratios of the isocyanate and polyol are 2:1, 3:1 and 4:1. The photoinitiator additive amounts are 0%, 5% and 15%. The signal-to-noise ratio (*S*/*N* ratio) formula in the Taguchi method is used to calculate the *S*/*N* ratio of the shear strength and peel strength. The main effect analysis and variance analysis are used to obtain the single-quality optimization design for shear strength and peel strength. Subsequently, the gray relational analysis method is used, with the *S*/*N* ratio of the shear strength and peel strength as the initial sequence, and the gray relational formula is used to generate two new sequences, Xi and Xj. The maximum value of the two sequences is selected as the reference sequence, and the rest are used as the comparison sequence. The difference between the sequences is calculated, and the gray relational coefficient and gray relational degree are calculated based on it. Finally, the main effect analysis is carried out on the gray relational degree, and the multiple quality optimization parameter design is obtained.

## 3. Results and Discussion

### 3.1. Chemical and Physical Analysis

#### 3.1.1. Fourier Transform Infrared Spectrometer (FTIR)

**Figure 5:**
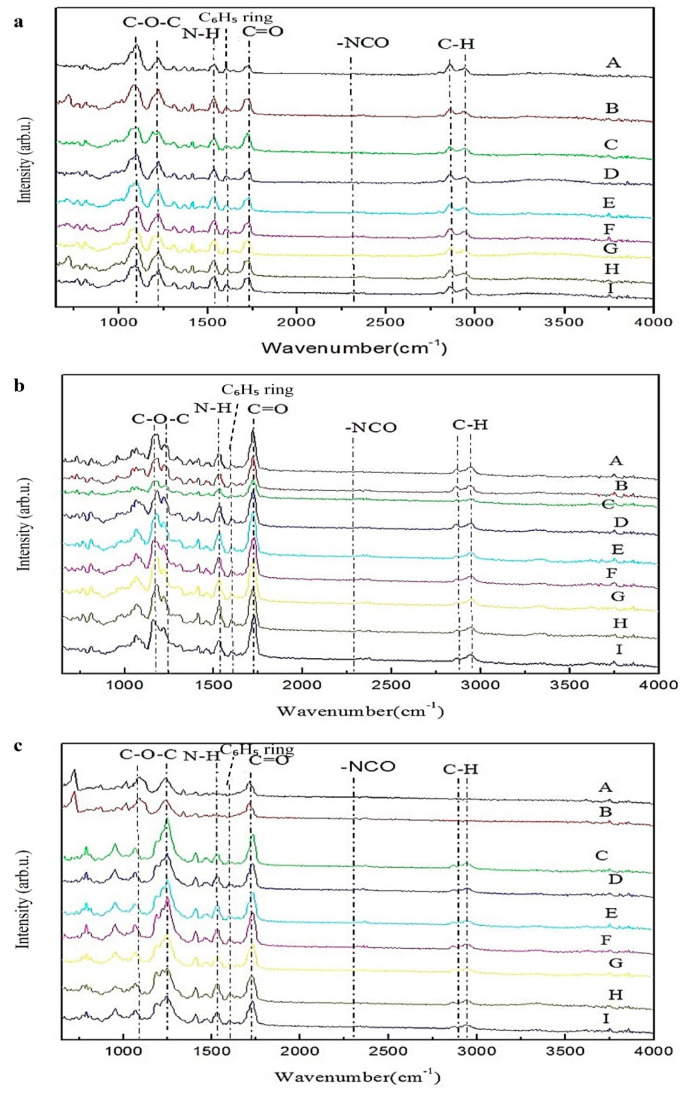
The FTIR spectra of dual-cure polyurethane prepared using (**a**) PTMG polyol, (**b**) PCL diol and (**c**) PHCD polyol (PTMG R2-0, R3-5, R4-15 as A, B, C), PCL R2-0, R3-5, R4-15 as D, E, F), and PHCD R2-0, R3-5, R4-15 as G, H, I).

The Fourier transform infrared spectroscopy (FTIR) instrument, a Digilab model (FTS -1000, Oxford, UK), was used to perform a functional group analysis on the synthesized dual-curing polyurethane hot-melt adhesive, as shown in Figure 5. When the beam contacts the sample (through transmission or reflection), different interference patterns are formed on the detector, which are then transformed via a Fourier transform to produce an infrared absorption spectrum. This mainly leads to energy changes between the vibrational and rotational levels of the substance caused by the absorption of infrared light. The measurement is commonly carried out by transmission, reflection and attenuated total reflection (ATR) methods. Generally, the wave number range of transmission infrared spectroscopy is 400~4000 cm^−1^, while the wave number range of attenuated total reflection infrared spectroscopy is 650~4000 cm^−1^. This study used an attenuated total reflection (ATR) mode for functional group identification, with an infrared wavelength range of 650~4000 cm^−1^ [24,25]. The FTIR spectra of the dual-curing polyurethane are shown in Figure 5 in order for the polyols used, PTMG, PCL and PHCD, respectively. Figure 5a–c represents the samples with a NCO:OH ratio of two, and they represent samples with NCO:OH ratios of three and four, respectively, with increasing ODA content from 0 to 15.

The peak at 1110 cm^−1^ corresponds to the C-O-C stretching vibration of the soft-segment diol; the peak at 1242 cm^−1^ corresponds to the stretching and bending vibrations of the isocyanate group; the peak at 1535 cm^−1^ indicates the presence of hydrogen bonding in the polyurethane; the peak at 1600 cm^−1^ corresponds to the phenyl ring in the isocyanate; the peak at 1730 cm^−1^ corresponds to the C=O stretching vibration of the isocyanate group, which increases with an increasing molar ratio of ODA; the peak at 2268 cm^−1^ corresponds to the NCO functional group of the isocyanate, which disappears due to a complete reaction during end-capping; and the peaks at 2680 cm^−1^ and 2940 cm^−1^ correspond to the stretching vibrations of CH_2_ and CH_3_, respectively [26]. The detailed peak values and functional groups are shown in Table 4.

#### 3.1.2. Advanced Polymer Chromatography (APC)

Advanced Polymer Chromatography (APC) was used in this study to analyze the molecular weight and molecular weight distribution of the polymers. The sample was dissolved in tetrahydrofuran (THF) and injected into a chromatography column with an internal diameter of 3 μm. When the sample solution flowed through the chromatography column, larger molecules were excluded from the pores and could only pass through the particle interstices, resulting in a faster rate. Smaller molecules fell into the pores and passed through at a relatively slower rate. After passing through a certain length of the chromatography column, the molecules were separated based on their relative molecular weight, with those with a larger relative molecular weight flowing out and being detected first, while those with a smaller relative molecular weight flowed out more slowly. Therefore, the molecular weight of the sample could be detected based on this principle. In this study, the sample was prepared by mixing it with a THF solvent in a ratio of 2 mg:1 mL. After the sample was completely dissolved, it was filtered using a 0.2 μmol syringe filter to avoid blockage of the pipeline. The Advanced Polymer Chromatography (APC) system automatically calculates Mn, Mw and the PDI based on the chromatographic separation of the sample [27].

**Figure 6:**
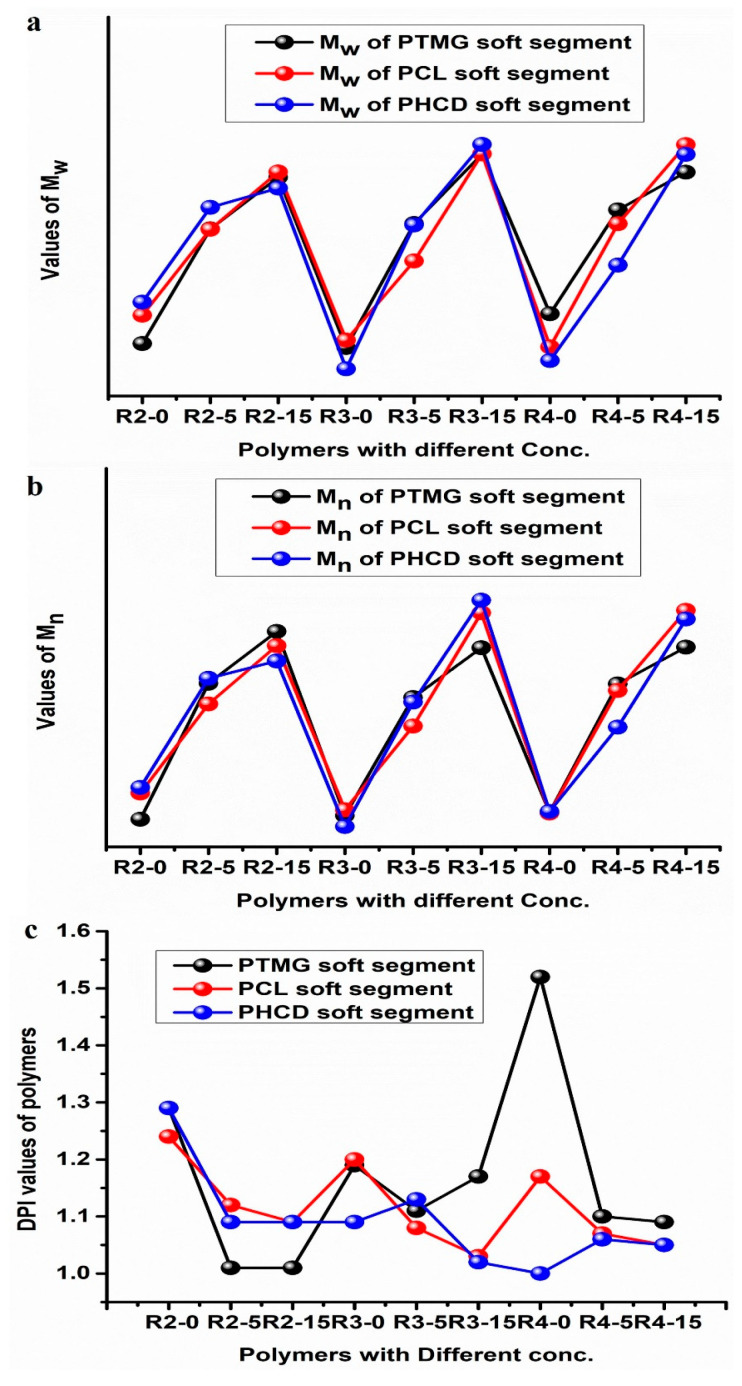
Molecular weight distribution at different NCO:OH, ratios and mol ratios of photopolymerizable monomers in polyol. (**a**) Average weight (M_w_) of PTMG, PCL and PHCD soft segments; (**b**) average number (M_n_) of PTMG, PCL and PHCD soft segments; and (**c**) DPI of PTMG, PCL and PHCD soft segments.

The molecular weight distribution of dual-cure polyurethane hot-melt adhesives was analyzed by APC under the same polyol, different R values and different amounts of photopolymerizable monomers [28], and the molecular weight distribution is shown in Figure 5. The sample names consist of two numbers, representing the NCO:OH, ratio value and the molar ratio of the added photopolymerizable monomers. It can be observed in Figure 6a,b that the use of soft-segment polyols causes a steady increase in the average number (M_n_) and average weight (M_w_) of dual-cure polyurethane hot-melt adhesives with the addition of photocurable monomers. Taking R4 as a standard, the Mn and Mw of the PTMG soft segment increase from 11,888 to 33,086 and 14,185 to 36,142; the PCL soft segment increases from 11,804 to 37,801 and 17,816 to 39,666; and the PHCD segment increases from 12,011 to 36,702 and 19,495 to 38,403. Moreover, the polydispersity index (PDI) of all the polymers is less than two, as can be observed in Figure 6c. In this study, 2-HEA was first used to end-cap the prepolymers, and it was observed that the polymerization degree of each sample without the addition of photocurable monomers is stable at around 10,000, but when ODA is added, the molecular weight can steadily increase by 10,000–20,000, achieving the effect of further growth after end-capping.

#### 3.1.3. Thermogravimetric Analysis (TGA)

The thermogravimetry analysis (TGA, TA Instruments-DuPont Q500, New Castle, DE, USA) was used to measure the weight loss and thermal decomposition temperature of a dual-cured polyurethane hot-melt adhesive at a specific temperature. The main principle is to place the sample in a heating furnace that can be heated, cooled or maintained at a constant temperature and introduce a fixed gas (nitrogen or oxygen) [29]. When the temperature rises to the decomposition temperature, evaporation temperature or oxidation temperature of a component in the sample, the sample will undergo weight loss due to decomposition, evaporation or oxidation, allowing for the determination of the thermal stability, decomposition temperature, component ratio, sample purity, moisture content and antioxidant properties of the material. The instrument was calibrated by placing an empty pan on it and zeroing the state after subtracting the weight of the empty pan. The sample weight was measured, and it should be less than 10 mg. The sample was placed in the instrument, and the parameter conditions were set from room temperature to 600 °C with a heating rate of 20 °C/min [30]. The weight loss of the dual-cured polyurethane hot-melt adhesive during heating was detected, and the test data will serve as a reference for future applications in temperature-stable conditions during processing. Nitrogen was chosen as the environmental gas in this study, and the heating condition was from room temperature to 600 °C at a heating rate of 20 °C/min [31,32]. This study analyzed the thermal stability of the photocured polyurethane hot-melt adhesive under a nitrogen atmosphere. Table 5 summarizes all the TGA data, with T5 and T10 indicating the temperatures at which 5% and 10% weight loss occurred [33], respectively. Figure 6 shows the TGA curves for different NCO:OH ratios, types of polyols and ODA addition levels.

**Figure 7:**
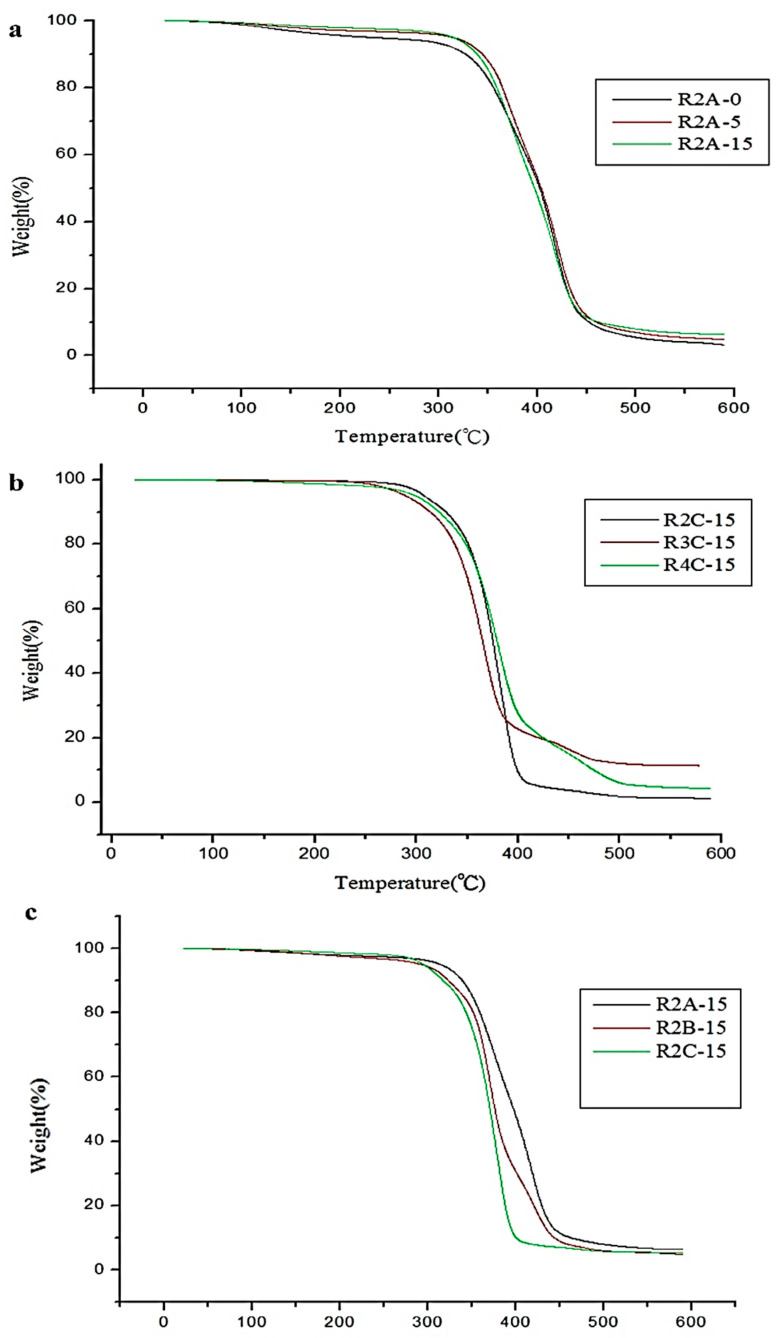
TGA curves of polyurethane hot-melt adhesives with the (**a**) same PTMG polyol and NCO:OH ratio, (**b**) the same PHCD polyol and ODA at different molar ratios and (**c**) the same NCO:OH ratio and different ODA contents.

Table 5 and Figure 7a–c show that before the temperature reaches 250 °C, the thermal weight loss of all dual-curing hot-melt adhesives does not exceed 5%. It is also observed that the thermal decomposition of polyurethane hot-melt adhesives can be divided into two stages: the first stage involves weight loss in the hard-chain segment area between 200 °C and 400 °C, and the second stage involves weight loss in the soft-chain segment between 400 °C and 500 °C. The thermal decomposition temperature of the polymer is affected by the heat resistance of the monomer used in the polymerization process. Therefore, in this study, 2-HEA and photopolymerizable monomers were used to introduce the double-bond functional groups into the dual-curing polyurethane hot-melt adhesives. Since ODA can not only react with end-capping 2-HEA but can also crosslink with the main chain of polyurethane to form a crosslinked structure, Table 6 shows that with the increase in the ODA addition, not only the number of double-bond functional groups but also the molecular weight of the polymer increased. Taken together, these two points can effectively enhance the thermal decomposition temperature of the dual-curing polyurethane hot-melt adhesive. When using the same type of polyol, it can be seen that the closer the NCO:OH, ratio is to one, the higher the thermal resistance temperature. Furthermore, compared to the other two types of polyols, the ether-based polyol has the highest thermal decomposition temperature.

### 3.2. Mechanical Properties of Hot-Melt Adhesives

The mechanical properties (peel strength and shear strength) of the hot-melt adhesive, by employing the Taguchi method with different polyols, R values and ODA contents of the dual-curing polyurethane hot-melt adhesive, were analyzed, and nylon fabric was used as the experimental substrate. The seamless bonding of the dual-curing polyurethane hot-melt adhesive to the nylon substrate was carried out with a UV exposure time of 2 s, a 100 °C processing temperature, a 1 min processing time and a 1 kg/cm^2^ processing pressure. It can be observed in Table 6, owing to the addition of 2-HEA as the end-capping agent, that the molecular weight was controlled at around 10,000, resulting in a decrease in the peel and shear strength, while both the peel and shear strength were improved by the addition of ODA as a crosslinking growth agent. It was noted that the soft segment of the polyurethane hot-melt adhesives reacted with polycarbonate > ester > polyether in the polyol. It was observed that when 15 moles of ODA as the crosslinking growth agent were added, the hot-melt adhesive showed the highest peel and shear strength, which ultimately affected the mechanical strength. The addition of 15 moles of ODA not only effectively controls the increase in molecular weight to 10,000–20,000 but also increases the C=O functional group on the molecular chain. These C=O functional groups bonded with the -NH groups of nylon through Wan der Waals (hydrogen bonds), allowing the dual-curing polyurethane hot-melt adhesive to tightly bond with the nylon fabric, which ultimately enhanced the mechanical strength.

### 3.3. Optimal Analysis of Dual-Cure Polyurethane Hot-Melt Adhesive Single-Quality Design

In this study, poly-alcohol (PTMG, PCL, PHCD) as A, the NCO:OH ratio (2:1, 3:1, 4:1) as B and the ODA mole ratio (0, 5, 15) as C were selected as the control factors for the experiment, respectively. The three control factors and their levels were inputted into the Taguchi L_9_ orthogonal array. The quality characteristics of peel strength and shear strength were analyzed using the signal-to-noise ratio (*S*/*N*) approach. The impact of each control factor at different levels on the quality characteristics of the dual-cure polyurethane hot-melt adhesive was evaluated. Finally, through confirmation experiments, a reliable, optimal single-quality parameter configuration was obtained.

#### 3.3.1. Single-Quality Optimization Analysis of Shear Strength

**Figure 8:**
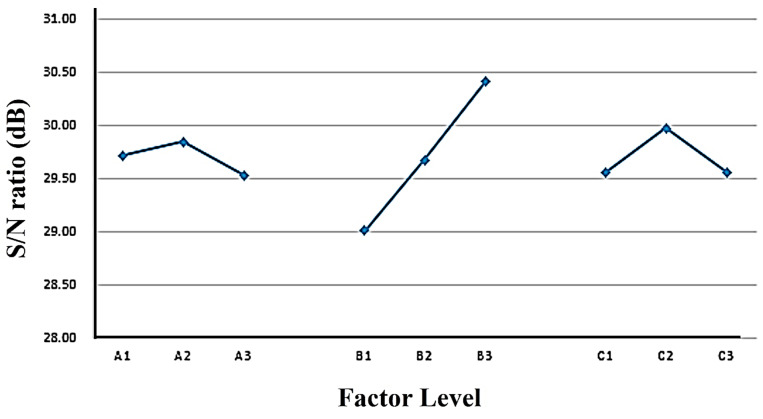
Factor response graph for shear strength of dual-cure polyurethane hot-melt adhesive.

The maximum desirable value of shear strength was obtained through the analysis to calculate the *S*/*N* ratios. After that, a main effect analysis and an ANOVA were conducted. Since a higher shear strength is desired, the larger-the-better *S*/*N* formula was used to calculate the *S*/*N* ratios. The results of the shear strength analysis, along with the mean and standard deviation, are given in Table 7. The response values for each factor were obtained for the nine experiments in terms of the shear strength after the *S*/*N* ratio calculation. The influence of each control factor at different levels on the dual-curing polyurethane hot-melt adhesive is determined and ranked by creating a response table and a response graph for the main effects analysis, as shown in Table S7 (Supplementary Document) and Figure 8. From these data, the optimal parameter ratio for the shear strength of the dual-curing polyurethane hot-melt adhesive was determined. The ANOVA results for the influence of each control factor on the double-cured polyurethane hot-melt adhesive are shown in Table S8 (Supplementary Document). The ANOVA was needed after the main effect analysis was completed for further confirmation of the significance and magnitude of the effect of each control factor [34]. The ANOVA showed that three important factors which affect the shear strength with F-values are, accordingly, the NCO:OH ratio, with an F-value of 362.33, the photocurable monomer, with an F-value of 42.37, and the polyol, with an F-value of 18.11 (if the F-value > 5, it is important, but if the F-value < 5, it is not important), which is consistent with the results obtained from the main effect analysis. The optimal combination for peel strength is NCO:OH, ratio of 4:1 and a 5 mol% addition of photocurable monomer, as A2, B3 and C2 are polyester polyols, respectively, according to the Taguchi analysis method. For this purpose, the total average of the *S*/*N* ratio for the nine experiments needs to be calculated. The average value is calculated as follows:

(8) $$\bar{\eta x}=\frac{1}{9}\;\sum_{n=1}^{9}\eta x\;=\;\frac{1}{9}\;(28.87\;+\;30.00\;+\;30.25\;+\ldots \ldots .+\;30.50\;+\;30.79)\;=\;29.69$$

Table 7 shows the averages of the three significant control factors. The optimal main effect analysis values are given in Table 6, and they are calculated by using the following method:

(9) $$S/N=\bar{\;\eta x}+(A2-\bar{\eta x})+(B3-\bar{\eta x})+(C2-\bar{\eta x})$$

*S*/*N* = 29.69 + (29.84 − 29.69) + (30.40 − 29.69) + (29.97 − 29.69) = 30.83 (10)

To make an effective estimation of the *S*/*N* ratio in the confirmation experiment, the 95% confidence interval (CI) needs to be calculated by using the following method:

(11) $${\mathrm{CI}}_{S/N}=\sqrt{F\alpha 1,v2\times \mathrm{Var}e\times (\frac{1}{n\mathrm{eff}}+\frac{1}{r})}$$

(12) $${\mathrm{CI}}_{S/N}=\sqrt{18.51\times 0.11\times \left(\frac{1}{\frac{9}{1+6}}+\frac{1}{3}\right)}=1.4962$$

where Fα1 and v2 are the F-distribution values of the significant control factors, α is the confidence level, v2 is the error variance degrees of freedom, V_are_ is the combined error variance, η_eff_ is the effective number of observations, and r is the number of analyses for the confirmation experiment. The CI_S/N_ value was 1.4962, and the confidence interval was 29.3338~32.3262 for the optimization analysis of the peel strength of the dual-curing polyurethane hot-melt adhesive.

This *S*/*N* ratio (30.62) indicated the high accuracy and good reproducibility of the experiment because it fell within the 95% confidence interval. Therefore, polyester polyol at a 4:1 NCO:OH, ratio and five molar additions of ODA were selected for the experiment’s confirmation. The shear strength results are shown in Table S9 (Supplementary Document).

#### 3.3.2. Optimization Analysis for Peel Strength Single-Factor Quality

**Figure 9:**
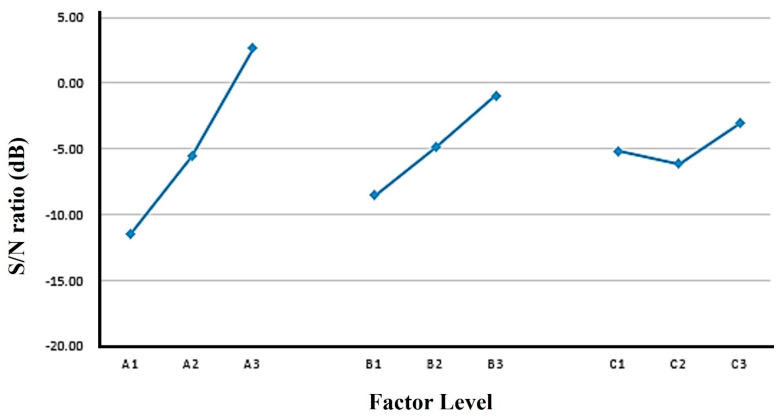
Factor response chart for peel strength of dual-cured polyurethane hot-melt adhesive.

The *S*/*N* ratios were calculated for each experimental group using the obtained results from the peel strength analysis through the use of LTB characteristics [34], and they are presented in Table 8. The response table and response plot of the main effect analysis are shown in Table 9 and Figure 9, respectively. This analysis provides insight into the degree to which each control factor affects the double-curing polyurethane hot-melt adhesive at different levels and ranks them. The optimal parameters for the peel strength optimization of the double-curing polyurethane hot-melt adhesive are identified. The response graph and response table showed that A3B3C3 had the highest factor levels to influence each control factor on the double-curing polyurethane hot-melt adhesive. To further confirm the magnitude of the impact of each control factor and determine its significance, an ANOVA was performed as well. The variance analysis results are shown in Table S10 (Supplementary Document).

The Taguchi analysis revealed that the optimal combination for peel strength is A3B3C3, which is composed of polycarbonate polyol in a NCO:OH ratio of 4:1 and 15 mole ratios of the photopolymerization monomer, respectively. Confirmation experiments were necessary and were conducted on the optimal parameter combination obtained. The overall average of the *S*/*N* ratio for the nine experiments is:

(13) $$\bar{\eta x}=\frac{1}{9}\;\sum_{n=1}^{9}\eta x\;=\;\frac{1}{9}\;(1.03\;+\;1.65\;+\;4.13\;+\ldots .+\;1.72\;+\;8.43)=\;-4.783$$

The predicted optimal *S*/*N* ratio needs to be determined by using the total average and significant control factors. According to Table S11, (Supplementary Document) there is only one significant control factor, and the calculation method is as follows:

(14) $$S/N=\bar{\eta x}+(A3-\bar{\eta x})+(B3-\bar{\eta x})+(C3-\bar{\eta x})\;=\;8.196$$

In order to make an effective estimation of the actual S/N ratio used to calculate the 95% confidence interval (CI) value, the following calculation was used:

(15) $${\mathrm{CI}}_{S/N}\;=\sqrt{F\alpha 1\times Vare\times \left(\frac{1}{neff}+\frac{1}{r}\right)\;}$$

(16) $${\mathrm{CI}}_{S/N}\;=\sqrt{18.51\times 1.02\times \left(\frac{1}{\frac{9}{1+6}}+\frac{1}{3}\right)\;}=\;4.5779$$

The F distribution value for the significant control factor is 18.51, and from Table S8 (Supplementary Document), the combined error degree of freedom is 2 with a 1.02 variance. The confidence interval for the optimal analysis of the peel strength of the double-cured polyurethane hot-melt adhesive is 3.6181 to 12.7739. Therefore, the confirmation experiment was conducted by using polycarbonate diol in a 4:1 NCO:OH, ratio and 15 mol% of ODA, which showed high accuracy and good experimental reproducibility, as shown in Table S9 (Supplementary Document), for the shear strength results. The obtained data were used to calculate the S/N ratio of the peel strength as 4.21, which falls within the 95% confidence interval, showing that the experiment has good accuracy and reproducibility.

### 3.4. Multi-Quality Optimization Analysis

The gray relational analysis method was used to perform multi-quality optimization parameter design for the dual-cure polyurethane hot-melt adhesive to optimize the peel strength and shear strength of the adhesive. Table 10 shows the signal-to-noise ratios of the nine groups in the analysis of the dual-curing polyurethane hot-melt adhesive for the gray relational generation. Table 11 shows the maximum value (4.03), the minimum value (−16.62) and the difference value (20.65) of the peel strength and the maximum value (30.50), the minimum value (28.75) and the difference value (1.76) of the shear strength. As this study aims to maximize the values of these two quality characteristics, the maximum characteristic found was used for the calculation. A gray relational generation was conducted to obtain a gray relational sequence. After the calculation, the gray correlation generation table was completed, and the next step was to calculate the difference between the reference sequence and each other sequence to obtain a sequence of differences, which is then plotted in Table S12 (Supplementary Document).

**Figure 10:**
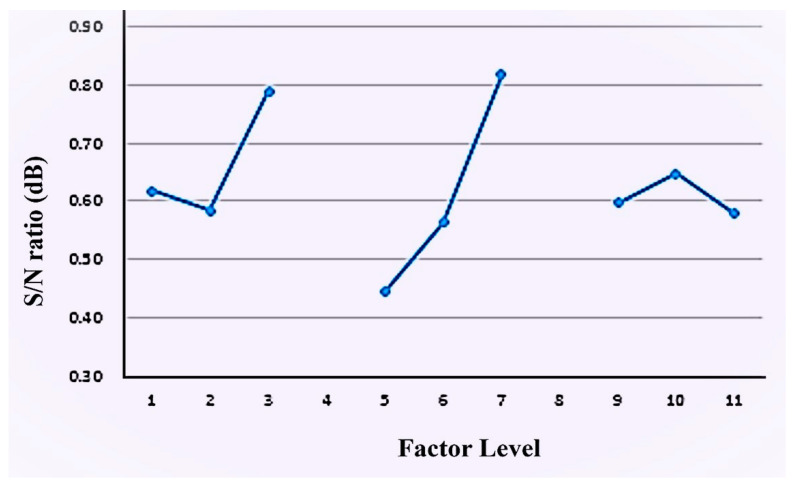
The main effect response plot of the dual-cure polyurethane hot-melt adhesive.

After calculating the difference between the sequences, the gray correlation degree values were used to determine the shear strength and peeling strength. The detailed values are shown in Table 12. After carrying out the gray relational analysis, the next step was to perform a main effect analysis on the gray relational degrees to obtain the final optimal design parameters for the multi-quality optimization. Therefore, the gray relational degree values are plotted into a main effect response, shown in Table 13, and a response plot in Figure 10, respectively. From these, the optimal design parameters for the dual-cured polyurethane hot-melt adhesive can be obtained. According to the response values in Table 13, the optimal levels for the multi-quality optimization of the dual-curing polyurethane hot-melt adhesive are for the combination A3B3C2, which means PHCD was selected as the soft-chain segment and a 4:1 NCO:OH, ratio was used with five molar ratios of ODA for polymerization. As shown in Table 13, the ranking of the main effect differences is B, A, then C, indicating that the ratio of R values is the most influential control factor, followed by the polyol, and finally the molar ratio of photocurable monomers. To verify the accuracy of the multi-quality optimization design obtained by the gray relational analysis method, experiments and analyses need to be conducted based on the control factors and the levels of the experimental parameters designed by the multi-quality optimization, and the enhancement effect of the mechanical strength obtained needs to be confirmed. The experimental results are shown in Tables S13 and S14 (Supplementary Document). After experiments, the results indicated that the dual-cured polyurethane hot-melt adhesive has an average shear strength of 34.94 and an average peel strength of 1.68 after the multi-quality optimization parameter design using gray correlation. Both qualities are within the 95% confidence interval of the single-quality optimization (shear strength confidence interval: 29.3338~32.3262; peel strength confidence interval: 3.6181~12.7739), which confirms the accuracy and reproducibility of the optimization parameters obtained through the gray correlation analysis.

### 3.5. Comparison with Commercial Hot-Melt Adhesives

The optimal parameters for the gray correlation-based multi-quality optimization were polycarbonate polyol with a NCO:OH ratio of 4:1 and a photocurable monomer mole ratio of five. Therefore, the confirmation experiments using this experimental design were compared with the non-optimized hot-melt adhesive and commercially available hot-melt adhesives in terms of their physical properties, and the results are shown in Tables S15 and S16. According to Table S15, the multi-quality optimized hot-melt adhesive had a peel strength (1.69 kg/cm) and shear strength (35.62 kg/cm^2^) which were 7.64 percent and 7.25 percent better than those of the unoptimized sample, respectively. The analysis demonstrated that the experimental parameters could be successfully optimized by using a multi-quality optimization design. This study required an evaluation of processing temperature, duration and pressure because seamless bonding can lead to different faults in the fiber if the temperature is too high. Table S16 showed that the processing temperatures of the commercial hot-melt adhesive and the hot-melt adhesive generated through multi-quality optimization were 100 °C and 150–170 °C, respectively, lowering the softening temperature by 33% to 41%. The hot-pressing pressure for the commercial hot-melt adhesive and multi-quality optimization were 15 psi and 40–60 psi, respectively, which was decreased from 63% to 75%. The lowering of the softening temperature, processing time (s) and hot-pressing pressure (psi) under synthesis conditions efficiently sped up the production of the synthesized material, decreasing labor and processing costs.

## 4. Conclusions

In this study, a dual-curing polyurethane hot-melt adhesive was prepared by using end-capping and UV-curing technology and applied to the seamless bonding technology of nylon fiber fabrics. The experimental parameter design was constructed using the L9 orthogonal table in the Taguchi quality engineering method, and the multi-quality optimization of experimental parameter planning was achieved by a gray correlation analysis. The soft-chain segment of polyurethane and the hard segments were used to polymerize the polyurethane prepolymer. The FTIR results showed the successful termination of the prepolymer’s polymerization reaction. The peak energy was at 1730 cm^−1^, indicating that the photopolymerizable monomers effectively bonded to the main chain. The APC results showed the termination of hydroxyethyl acrylate in the polymerization reaction. It was also observed that under the addition of octyl decyl acrylate and exposure to UV irradiations, the molecular weight increased from 10,000 to 20,000, which indicated the conversion of the secondary growth of the molecular chain from photopolymerizable monomers. The TGA results showed that after the molecular weight and main chain grew with the help of octyl decyl acrylate, the thermal decomposition temperature of the polyurethane hot-melt adhesive also increased due to the addition of double-bond functional groups, and the thermal weight loss before 250 °C did not exceed 5%. It was observed that after adding hydroxyethyl acrylate and octyl decyl acrylate to the polyurethane hot-melt adhesive, the presence of C=O and N-H functional groups in their molecules may interact with the polar groups of the nylon substrate, thereby enhancing the adhesion between the substrate and the adhesive. The experimental parameters of the polyurethane hot-melt adhesive were optimized by employing the Taguchi quality engineering approach and a gray relational analysis. The most suitable set of parameters comprised a polycarbonate soft segment, a 4:1 NCO:OH ratio, and a five-mole addition of photopolymerizable monomer. The in-situ polymerization and two-step polymerization processes were utilized to generate the dual-curing polyurethane hot-melt adhesive, which was subsequently utilized to connect nylon fabrics. It had an average shear strength of 34.94 kg/cm^2^ and a peel strength of 1.68 kg/cm. It also showed higher performance in the peel and shear strength tests. To optimize the experimental parameter design and satisfy the need for seamless bonding in textile factories, goal values will be determined properly. This will allow for an effective increase in production speed and a decrease in processing time and costs as well.

## Figures and Tables

**Table 1:** Polymerization parameters of dual-cure polyurethane hot melt adhesive.

	Control Factors	Level Value 1	Level Value 2	Level Value 3
A	Polyol	PTMG	PCL	PHCD
B	NCO:OH ratio	2:1	3:1	4:1
C	ODA molar ratio	0	0.5	0.15

**Table 2:** Detailed formulation of samples for dual-curing polyurethane hot-melt adhesive.

Materials Names	MDI (Moles)	PTMG (Moles)	PCL (Moles)	PHCD (Moles)	2-HEA (Moles)	ODA (Moles)
R2-0ODA	4	2	0	0	4	0
R2-5ODA	4	2	0	0	4	5
R2-15ODA	4	2	0	0	4	15
R3-0ODA	6	0	2	0	8	0
R3-5ODA	6	0	2	0	8	5
R3-15ODA	6	0	2	0	8	15
R4-0ODA	8	0	0	2	12	0
R4-5ODA	8	0	0	2	12	5
R4-15ODA	8	0	0	2	12	15

**Table 3:** Taguchi L9 orthogonal array.

	Factors	A (Polyol Category)	B (NCO:OH Ratio)	C (ODA Molar Ratio)
Experiment				
1		1	1	1
2		1	2	2
3		1	3	3
4		2	1	2
5		2	2	3
6		2	3	1
7		3	1	3
8		3	2	1
9		3	3	2

**Table 4:** Corresponding functional groups and peak region values in the FTIR spectra of polyurethane hot-melt adhesives.

Functional Group	Wavenumber (cm^−1^)	Functional Group	Wavenumber (cm^−1^)
C-H	2860–2950 cm^−1^	Benzene ring	1590–1600 cm^−1^
NCO	2268 cm^−1^	N-H	1535 cm^−1^
C=O	1720–1735 cm^−1^	C-O-C	1100–1245 cm^−1^

**Table 5:** TGA data of dual-cure polyurethane hot-melt adhesives in nitrogen atmosphere.

Sample Name	T_5_ (°C)	T_10_ (°C)	Sample Name	T_5_ (°C)	T_10_ (°C)
PTMG soft segment			PCL soft segment		
R2-0	233.52	327.03	R3-5	295.54	322.80
R2-5	283.64	318.39	R3-15	302.22	324.53
R2-15	328.20	344.78	R4-0	285.29	312.87
R3-0	299.30	320.09	R4-5	295.29	321.67
R3-5	302.94	325.43	R4-15	304.68	330.38
R3-15	311.96	349.89	PHCD soft segment		
R4-0	263.84	313.42	R2-0	279.32	308.23
R4-5	293.07	320.89	R2-5	289.97	314.43
R4-15	295.29	321.67	R2-15	306.60	324.10
PCL soft segment			R3-0	292.82	318.23
R2-0	292.43	322.86	R3-5	293.08	311.31
R2-5	295.54	322.80	R3-15	299.66	323.98
R2-15	312.71	334.30	R4-0	291.03	318.32
R3-0	291.80	316.41	R4-5	302.78	322.98
R3-5	295.54	322.80	R4-15	304.46	326.62

**Table 6:** Analysis of peel strength, shear strength and softening point of dual-curing polyurethane hot-melt adhesives.

Sample Name		Softening Point (°C)	Peel Strength (kg/cm)	Shear Strength (kg/cm^2^)
Target value		>100	>0.39	>13
	PTMG			
R2-0		75	0.15	28.50
R3-5		75	0.23	31.77
R4-15		80	0.52	32.46
	PCL			
R2-5		65	0.34	29.67
R3-15		65	0.52	30.43
R4-0		55	0.81	33.11
	PHCD			
R2-0		70	1.13	27.34
R3-5		80	1.33	29.68
R4-15		80	1.59	33.71

**Table 7:** Analysis Results of Shear Strength of Dual-cured Polyurethane Hot-melt Adhesive.

No.	Factors								
	A	B	C	Test 1 (MPa)	Test 2 (MPa)	Test 3 (MPa)	Ave. (MPa)	St. dev.	S/N (dB)
1	1	1	1	28.5	27.06	27.84	27.80	0.72	28.87
2	1	2	2	31.77	31.46	31.69	31.64	0.16	30.00
3	1	3	3	32.46	32.64	32.58	32.56	0.09	30.25
4	2	1	2	29.67	29.37	29.48	29.51	0.15	29.40
5	2	2	3	30.43	30.67	30.27	30.46	0.20	29.67
6	2	3	1	33.11	33.31	33.48	33.30	0.19	30.45
7	3	1	3	27.34	27.02	27.8	27.39	0.39	28.75
8	3	2	1	29.68	29.19	29.03	29.30	0.34	29.34
9	3	3	2	33.71	33.21	33.62	33.51	0.27	30.50

**Table 8:** Results of peel strength analysis for dual-cure polyurethane hot-melt adhesives.

No.	SN (dB)								
	A	B	C	Test 1	Test 2	Test 3	Average	Standard	
1	1	1	1	0.15	0.13	0.18	0.15	0.03	−16.62
2	1	2	2	0.23	0.27	0.24	0.25	0.02	−12.25
3	1	3	3	0.52	0.54	0.55	0.54	0.02	−5.42
4	2	1	2	0.34	0.28	0.32	0.31	0.03	−10.20
5	2	2	3	0.52	0.61	0.57	0.57	0.05	−5.02
6	2	3	1	0.81	0.85	0.88	0.85	0.04	−1.47
7	3	1	3	1.13	1.17	1.17	1.16	0.02	1.26
8	3	2	1	1.33	1.38	1.36	1.36	0.03	2.64
9	3	3	2	1.59	1.57	1.61	1.59	0.02	4.03

**Table 9:** Factor response table for peel strength of dual-cure polyurethane hot-melt adhesives.

	A (kg/cm)	B (kg/cm)	C (kg/cm)
Level 1	−11.43	−8.52	−5.15
Level 2	−5.56	−4.87	−6.14
Level 3	2.64	−0.95	−3.06
Max	2.64	−0.95	−3.06
Min	−11.43	−8.52	−6.14
effect	14.07	7.57	3.08
Ranking	1	2	3

**Table 10:** Signal-to-Noise Ratio Sequence Table for Peel Strength and Shear Strength.

	Peel Strength S/N Ratio	Shear Strength S/N Ratio
1	−16.62	28.87
2	−12.25	30.00
3	−5.42	30.25
4	−10.20	29.40
5	−5.02	29.67
6	−1.47	30.45
7	1.26	28.75
8	2.64	29.34
9	4.03	30.50
Maximum value	4.03	30.50
Minimum value	−16.62	28.75
Difference	20.65	1.76

**Table 11:** Gray correlation generation table for peel strength and shear strength.

	Peel Strength S/N Ratio	Shear Strength S/N Ratio
X_0_	1	1
X_1_	0.07	0
X_2_	0.72	0.22
X_3_	0.86	0.54
X_4_	0.37	0.31
X_5_	0.53	0.56
X_6_	0.97	0.73
X_7_	0.00	0.87
X_8_	0.34	0.93
X_9_	1.00	1.00
Maximum value	1.00	1.00
Minimum value	0.00	0.00

**Table 12:** Gray correlation degree table for peel strength and shear strength.

	Peel Strength S/N Ratio	Shear Strength S/N Ratio	Average
X_0_	1.00	1.00	1.00
X_1_	0.33	0.35	0.34
X_2_	0.39	0.64	0.51
X_3_	0.52	0.78	0.65
X_4_	0.42	0.44	0.43
X_5_	0.53	0.51	0.52
X_6_	0.65	0.94	0.80
X_7_	0.79	0.33	0.56
X_8_	0.88	0.43	0.66
X_9_	1.00	1.00	1.00

**Table 13:** Main Effects Response Table for Dual-Cured Polyurethane Hot-melt Adhesive.

	A	B	C
	Polyol	NCO:OH Ratio	ODA Molar Ratio
Level 1	0.62	0.44	0.60
Level 2	0.58	0.56	0.65
Level 3	0.74	0.82	0.58
Max	0.74	0.82	0.65
Min	0.58	0.44	0.58
effect	0.15	0.37	0.07
ranking	2.00	1.00	3.00

## Data Availability

The data presented in this study are available on request from the corresponding author.

## Supplementary Materials

The following supporting information can be downloaded at: https://www.mdpi.com/article/10.3390/polym16040467/s1. List of Tables: Table S1. Basic Orthogonal Table for Taguchi Method; Table S2. Taguchi L9 Orthogonal Array; Table S3. Factor Effecting the response; Table S4. Illustration of Analysis of variance; Table S5. F-value Table for 95% Confidence Level; Table S6. objectives of this study to get the target; Table S7. Factor response table for shear strength of dual-cure polyurethane hot melt adhesive; Table S8. The ANOVA analysis table for shear strength of dual-curing polyurethane hot melt adhesive; Table S9. Experimentation of shear strength for Dual-Cure Polyurethane Hot Melt Adhesives; Table S10. ANOVA Analysis Table of Peel Strength for Dual-curing Polyurethane Hot Melt Adhesive; Table S11. Confirmation Experiment for Dual-Cure Polyurethane Hot Melt Adhesive Peel Strength; Table S12. Difference Sequence for Peel Strength and Shear Strength; Table S13. Confirmation experiment output table (peel strength) for dual-cure polyurethane hot melt adhesive; Table S14. Confirmation experiment output table (shear strength) for dual-cure polyurethane hot melt adhesive; Table S15. Comparison of properties between multi-quality experiment and commercial hot melt adhesive; Table S16. Comparison of processing conditions between optimized experiment and commercial hot melt adhesive; List of contents: S1.1. Signal to Noise Ratio; S1.2. Main Effects Analysis; S1.3. Analysis of variance; S1.4. Calculation of Grey Relational Analysis; S1.5. Industry requirements. List of Figures: Figure S1. (a–c) Quality Loss function of STB, LTB, and NTB characteristics.
