# Supplementary material for: Study of Synthesis of Dual-Curing Thermoplastic Polyurethane Hot-Melt Adhesive and Optimization by Using Gray Relational Analysis to Apply in Fabric Industry to Solve Seamless Bonding Issues
Source: Polymers (Basel). 2024 Feb 7;16(4):467. doi: 10.3390/polym16040467 (PMC10892432; doi:10.3390/polym16040467)
Supplement: Supplementary file 1 [file polymers-16-00467-s001.zip › polymers-2823919-supplementary.pdf]

Supplementary document

Study of synthesis of dual-curing thermoplastic polyurethane hot-melt adhesive and optimization by using Grey relational analysis to apply on fabric industry to solve seamless bonding issues

Sheng-Yu Lin, Naveed Ahmad, Chung-Feng Jeffrey Kuo*

Department of Materials Science and Engineering, National Taiwan University of Science and Technology, Taipei, 10607, Taiwan, ROC

Corresponding Author: Chung-Feng Jeffrey Kuo *, Email: jeffreykuo@mail.ntust.edu.tw

List of Tables

Table S1. Basic Orthogonal Table for Taguchi Method

Table S2. Taguchi L_9 (3^4) Orthogonal Array

Table S3. Factor Effecting the response

Table S4. Illustration of Analysis of variance

Table S5. F-value Table for 95% Confidence Level

Table S6. objectives of this study to get the target

Table S7. Factor response table for shear strength of dual-cure polyurethane hot melt adhesive

Table S8. The ANOVA analysis table for shear strength of dual-curing polyurethane hot melt adhesive

Table S9. Experimentation of shear strength for Dual-Cure Polyurethane Hot Melt Adhesives

Table S10. ANOVA Analysis Table of Peel Strength for Dual-curing Polyurethane Hot Melt Adhesive

Table S11. Confirmation Experiment for Dual-Cure Polyurethane Hot Melt Adhesive Peel Strength

Table S12. Difference Sequence for Peel Strength and Shear Strength

Table S13. Confirmation experiment output table (peel strength) for dual-cure polyurethane hot melt adhesive

Table S14. Confirmation experiment output table (shear strength) for dual-cure polyurethane hot melt adhesive

Table S15. Comparison of properties between multi-quality experiment and commercial hot melt adhesive

Table S16. Comparison of processing conditions between optimized experiment and commercial hot melt adhesive

List of contents

S1.1. Signal to Noise Ratio
S1.2. Main Effects Analysis
S1.3. Analysis of variance
S1.4. Calculation of Grey Relational Analysis
S1.5. Industry requirements

List of Figures

Figure S1. (a-c) Quality Loss function of STB, LTB, and NTB characteristics

Table S1. Basic Orthogonal Table for Taguchi Method

Orthogonal table	number of columns	Maximum number of factors	Maximum number of level rows			
			2 level number	3 level number	4 level number	5 level number
L_4	4	3	3			
L_8	8	7	7			
L_9	9	4		4		
L_{12}	12	11	11			
L_{16}	16	15	15			
L_{16}	16	5			5	
L_{18}	18	8	1	7		
L_{25}	25	6				6
L_{27}	27	13		12		
L_{32}	32	31	31			
L_{32}	32	10	1		9	
L_{36}	36	23	11	12		
L_{36}	36	16	3	13		
L_{50}	50	12	1			11
L_{54}	54	26	1	25		
L_{64}	64	63	63			
L_{64}	64	21			21	
L_{81}	81	40		40		

Table S2. Taguchi L_9 (3^4) Orthogonal Array

Experiment \ Factor	A	B	C
1	1	1	1
2	1	2	2
3	1	3	3
4	2	1	2
5	2	2	3
6	2	3	1
7	3	1	3
8	3	2	1
9	3	3	2

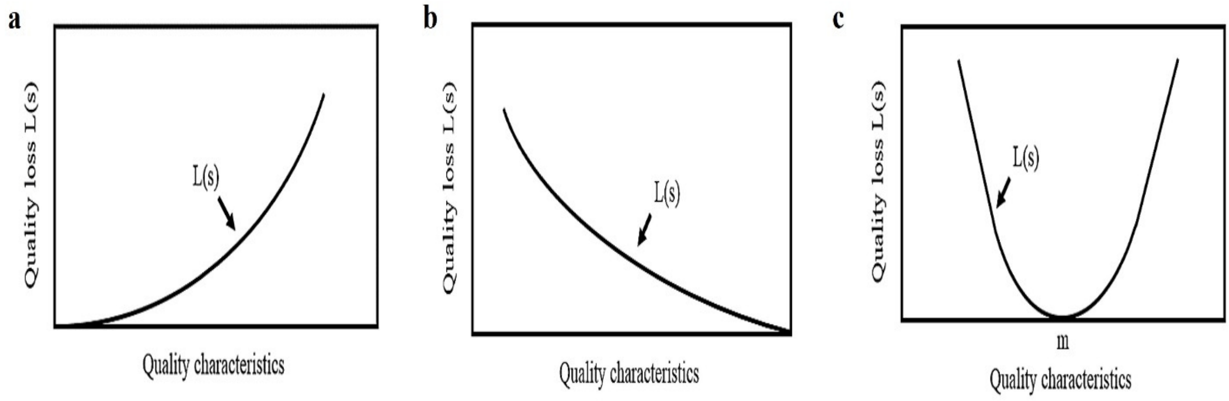

Figure S1. (a-c) Quality Loss function of STB, LTB, and NTB characteristic

S1.1. Signal to Noise Ratio

The Signal to Noise Ratio (SNR) used for the target value of the quality characteristic and the variation of the quality characteristic. The optimal SNR characteristics, such as, it can reflect the variability of the quality characteristic, predict the quality characteristic even when the target value

changes, measure relative quality. The SNR is measuring the quality of a product by using Taguchi Method's S/N ratio, described as

$$\chi = \log_{10} \frac{\text{Predictable beneficial variation}}{\text{Unpredictable adverse variation}} \quad \chi\text{=S/N ratio} \tag{S1}$$

A larger χ indicates better quality. The LTB type quality characteristic and its infinite or the maximum possible value, without adjustment factor. Its formula given as

$$S/N_{LTB} = -10 \log_{10} \left[\frac{1}{n} \sum_{i=1}^{n} \frac{1}{{y_i}^2}\right] \tag{S2}$$

The STB is a continuous non-negative variable, and the objective is to minimize both the variance and the mean value. Therefore, the ideal situation is when the value is zero, without any adjustment factor. The type and the formula are explained in equation

$$S/N_{STB} = -10 \log_{10} \left[\frac{1}{n} \sum_{i=1}^{n} {y_i}^2\right] \tag{S3}$$

The main characteristics of target value quality characteristic which is a continuous variable without negative values. The formula is shown as

$$S/N_{NTB} = 10 \log[\frac{\overline{y^2}}{S^2}] \tag{S4}$$

where, S represents the standard deviation during the experiment, and $\overline{y}$ represents the mean value during the experiment measurement.

S1.2. Main Effects Analysis

In main effect analysis, the main effect values of each factor are calculated to determine the size of the influence of each control factor on the quality characteristics of double-cured polyurethane hot melt adhesive's peel strength and shear strength which needs to be conducted through a universal testing machine. Based on peel strength and shear strength, the signal-to-noise ratio (S/N ratio) for each set of experimental parameters is calculated to establish a factor effect response table for the S/N ratio, as shown in Table S3. If factor A has Y levels, each level with S signal-to-noise ratios, the formulas for the average response value IY and difference ΔI for each level are shown below,

$$I_Y = \frac{1}{V} \sum_{f=1}^{V} \mu \tag{S5}$$

Where, μ is the signal-to-noise ratio of the factor at that level.

$$\triangle I = I_{YMAX} - I_{YMIN} \tag{s6}$$

Table S3. Factor Effecting the response

	I	J	K	L	M	N
Level 1	I_1	J_1	K_1	L_1	M_1	N_1
Level 2	I_2	J_2	K_2	L_2	M_2	N_2
Level 3	I_3	J_3	K_3	L_3	M_3	N_3
MAX	I_{MAX}	J_{MAX}	K_{MAX}	L_{MAX}	M_{MAX}	N_{MAX}
MIN	I_{MIN}	J_{MIN}	K_{MIN}	L_{MIN}	M_{MIN}	N_{MIN}
Difference	$\triangle_I$	$\triangle_J$	$\triangle_K$	$\triangle_L$	$\triangle_M$	$\triangle_N$

S1.3. Analysis of variance

The main purpose of ANOVA analysis is to investigate the correlation between the continuous dependent variable and independent variables. The ANOVA analysis focuses more on the contribution of each control factor to the improvement of experimental quality. Based on the F distribution, the importance of each factor can be estimated, providing a more objective way. In Taguchi quality engineering, ANOVA analysis relies on the F distribution as the basis for probability distribution. First, the sum of squares and degrees of freedom for each control factor is calculated. Then, these two values are used to calculate the variance (Var) and F value. Finally, the results are used to create an ANOVA table, as explained in Table S4.

Table S4. Illustration of Analysis of variance Table

Factor	SS	DOF	Var	F	ρ	P
A	SS_A	DOF_A	Var_A	F_A	ρ_A	
B	SS_B	DOF_B	Var_B	F_B	ρ_B	
C	SS_C	DOF_C	Var_C	F_C	ρ_C	
……	……	……	……	……	……	……
H	SS_H	DOF_H	Var_H	F_H		
error	SS_e	DOF_e	Var_e			
total	SS_T	DOF_T				

ANOVA analysis includes sum of squares, degrees of freedom, variance, F-distribution value, contribution, and P-value. The explanations for each item were given as

The sum of squares is divided into three types: total sum of squares, factor sum of squares, and error sum of squares.

Total sum of squares (SS_T) = $\sum_{r=1}^{n}(\delta r - \overline{\delta r})$ (S7)

In the equation, δr represents the S/N ratio for each experiment analysis, and ¯δr represents the average value of the S/N ratio for all experiment analyses.

SS control is the variance of each control factor. Assuming a control factor has r levels, and each level has t replicates of signal-to-noise ratio, the calculation for SScontrol were

SScontrol = t × $\sum_{n=1}^{r}(\overline{\delta rn} - \overline{\delta n})^2$ (S8)

The $\overline{\delta rn}$ in the equation represents the average value of the signal-to-noise ratio obtained for the control factor under the r levels.

The calculation of the error sum of squares is obtained by subtracting the sum of squares of all control factors from the total sum of squares, as shown in equation

$SSe = SS_T – SScontrol$ (S9)

The degree of freedom is a measure of the amount of information obtained. In statistics, it refers to the number of unrestricted independent variables when estimating a parameter using a statistical measure of a sample. Generally, the larger the degree of freedom, the more information can be obtained. In Taguchi's method, the degrees of freedom are divided into total degrees of freedom, control factor degrees of freedom, and error degrees of freedom.

Total degrees of freedom (DOF_T)

The total number of degrees of freedom is equal to the total number of experiments minus one.

$DOF_T = (m \times r) - 1$ (S10)

Control factor degrees of freedom ($DOF_{control}$)

The degree of freedom for a control factor is equal to the number of levels of the factor minus one.

$DOF_{control}$=number of levels -1 (S11)

Error degrees of freedom (DOFerror)

The value of error degrees of freedom is obtained by subtracting the degrees of freedom of control factors from the total degrees of freedom.

DOF error = $DOF_T - DOF_{control}$ (S12)

The mean square (MS), also known as variance, is the ratio of the sum of squares to degrees of freedom. Since the values in the experimental analysis are related to the sum of squares, to reduce the influence of these two values on the mean square, we divide the sum of squares by the corresponding degrees of freedom to obtain the variance, which is the mean square, as shown in Equation (3-15). Additionally, the mean square error is calculated using the following equations

$$\mathrm{Var}_{f} = \frac{\mathrm{SScontrol}}{\mathrm{DOFcontrol}} \tag{S13}$$

$$\mathrm{Var}_{e} = \frac{\mathrm{SSe}}{\mathrm{DOFerror}} \tag{S14}$$

F-value is a numerical value that describes the F-distribution and indicates the relationship between error variance and factor effects. It can be used to evaluate the impact of each control factor on the quality characteristics of the experiment.

$$\mathrm{F} = \frac{Varf}{Vare} \tag{S15}$$

The contribution in Taguchi quality engineering refers to the relative ability of a control factor to reduce variability, and it can be used to determine the proportion of total sum of squares and calculated by using the following formula

$$\text{Percent contribution } (\rho) = \frac{\mathrm{SScontrol}}{\mathrm{SST}} \times 100\% \tag{S16}$$

P-value is a measure of the probability used in experimental analysis.

The confidence level of the control factor can be calculated through the P-value, and the larger the confidence level, the more important the factor is to the quality characteristic. The formula for the confidence level is shown

$$\text{Confidence level} = (1\text{–}\mathrm{P}) \times 100\% \tag{S17}$$

In statistics, a confidence interval (CI) for a sample of data from a population is an estimation of an unknown population parameter using an interval, which includes information about the estimation accuracy. The confidence interval is a way to express the error associated with estimating an unknown value (e.g., the signal-to-noise ratio for each experiment in Taguchi method) as a probability that the unknown value falls within a certain range. To verify the reliability and rationality of Taguchi parameter design, experimental confirmation is required. First, the signal-to-noise ratio is predicted using the additive model under the optimal factor configuration conditions, followed by calculating the confidence interval of the optimized configuration based on the estimated signal-to-noise ratio. Finally, the experimental signal-to-noise ratio is compared with the estimated confidence interval. If the experimental signal-to-noise

ratio falls outside the confidence interval or deviates significantly from it, it indicates that there may be a problem with the experimental design, and the control factors and levels need to be reelected. It is also necessary to consider whether other factors may have affected the experimental results, and further improvements to the experiment and parameters should be made until good experimental reproducibility is achieved. The formula for predicting the optimal configuration signal-to-noise ratio SN is shown in Equation

$$SN = \overline{\eta r} + \sum_{r=1}^{n}(\eta s - \overline{\eta r}) \tag{S18}$$

Where, $\overline{\eta r}$ represents the average value signal-to-noise ratios, ηs is the signal-to-noise ratio of significant control factors, and n is the number of significant factors. To effectively estimate the signal-to-noise ratio of each experiment, the confidence interval of the confirmation experiment for the optimal control factor pairing of the single quality optimization quality characteristic needs to be calculated. The formula for calculating the confidence interval of the confirmation experiment is shown as equation

$$CI_{S/N} = \sqrt{F_{\alpha;1,v2} \times Var_e \times \left(\frac{1}{n_{eff}} + \frac{1}{r}\right)} \tag{S19}$$

Where, $F_{\alpha:1}$ is the F-distribution value of significant control factors, α is the confidence level, V_2 is the degrees of freedom of combined error variance, Var_e is the variance of combined error, r is the number of measurements in the confirmation experiment, and neff is the effective analysis value, which is calculated by the formula as follows.

$$\eta_{eff} = \frac{Total\ number\ of\ Experimental\ groups}{1 + (Degree\ of\ freedom\ of\ significant\ figures)} \tag{S20}$$

Regarding the significance level α, currently in statistics and calculations, most commonly use 0.05, which means that the confidence level of the confirmation experiment can be as high as 95%. Therefore, the F distribution value for the significance level can be found in Table S5.

This table can be used to look up the F value for a 95% confidence level. For example, F (0.05; 2, 10) = 4.10 means that at a 95% confidence level, the significant factor F value is 4.10 under the condition of combined error degrees of freedom being 10. The reliability and reproducibility of the experimental design can be verified by comparing the calculated confirmation experiment S/N ratio (S/Nv) with the confidence interval obtained from the significant factor's F value. The verification formula is shown as follows

$$SN - CI_{S/N} \leq S/Nv \leq SN + CI_{S/N} \tag{S21}$$

Table S5. F-value Table for 95% Confidence Level

DOF_e	1	2	3	4	5
1	161.5	199.5	215.7	224.6	230.2
2	18.51	19.00	19.16	19.25	19.30
3	10.13	9.95	9.28	9.12	9.01
4	7.71	6.94	6.59	6.39	6.26
5	6.61	5.79	5.41	5.19	5.05
6	5.99	5.14	4.76	4.53	4.39
7	5.59	4.74	4.35	3.84	3.69
8	5.32	4.46	4.07	3.63	3.48
9	5.12	4.26	3.86	3.48	3.33
10	4.96	4.10	3.71	3.36	3.20
11	4.84	3.98	3.59	3.26	3.11
12	4.75	3.89	3.49	3.18	3.03
13	4.67	3.81	3.41	3.11	2.96
14	4.60	3.74	3.34	3.06	2.90
15	4.54	3.68	3.29	3.01	2.85
16	4.49	3.63	3.24	2.96	2.81
17	4.45	3.59	3.20	2.93	2.77
18	4.41	3.55	3.16	2.90	2.74
19	4.38	3.52	3.13	2.87	2.71
20	4.35	3.49	3.10	2.84	2.68
21	4.32	3.47	3.07	2.82	2.66
22	4.30	3.44	3.05	2.80	2.64
23	4.28	3.42	3.03	2.78	2.62
24	4.26	3.40	3.01	2.78	2.62
25	4.24	3.39	2.99	2.763	2.60

S1.4. Calculation of Grey Relational Analysis

Grey Rational Analysis used to perform the conversion during grey correlation generation, and the corresponding calculation method adopted which was based on the different expected values of each column and values between 0 and 1. The following equations given as:

Favorable property of larger values (higher expected target values are preferred)

$$Xi^*(k) = \frac{Xi(k) - \min Xi(k)}{\max Xi(k) - \min Xi\ (k)} \tag{S22}$$

Favorable property of smaller values (lower expected target values are preferred)

$$Xi^*(k) = \frac{\max Xi(k) - Xi(k)}{\max Xi(k) - \min Xi\ (k)} \tag{S23}$$

Target Value Feature (where the expected target value is a specific value, represented by OT)

$$Xi^*(k) = \frac{|Xi(k) - OT|}{\max\{\max Xi(k) - OT, OT - \min Xi(k)\}} \tag{S24}$$

Where, Xi*(k) represents generated gray correlation value, max Xi(k) represents maximum value, and min Xi(k) represents the minimum value from the original sequence and the calculation of grey relational coefficient is performed by first selecting a reference sequence X0(k), while the other sequences are considered as comparison sequences (X1(k), X2(k), ..., Xp(k)). The formula for calculating grey relational coefficient is as follows：

$$\gamma 0p\ (k) = \frac{\min(p)\min(k)|X0(k) - Xp(k)| + \zeta \max(p)\max(k)|X0(k) - Xp(k)|}{|X0(k) - Xp(k)| + \zeta \max(p)\max(k)|X0(K) - Xp(k)|} \tag{S25}$$

The term min(p)min(k)|X0(k)-Xp(k)| represents the minimum absolute difference between X0(k) as the reference sequence and Xp(k) as the comparative sequence. Similarly, the term max(p)max(k)|X0(k)-Xp(k)| represents the maximum absolute difference between X0(k) as the reference sequence and Xp(k) as the comparative sequence. Therefore, the formula can be simplified as follows：

$$\gamma 0p\ (k) = \frac{\Delta \min + \zeta \Delta max}{\Delta\ 0i(k) + \zeta \Delta max} \tag{S26}$$

where, Δ0i(k) is difference represented the absolute difference between the reference sequence and the comparison sequence, and Δmax and Δmin represent the maximum and minimum values among them. ζ is the discrimination coefficient, with ζ∈ [0,1], Typically, the discrimination coefficient is set to 0.5. The changing the value of discrimination coefficient affects the numerical value of the grey correlation coefficient. After obtaining the gray correlation

coefficients of different sequences, the average value of the gray correlation coefficients is usually used as the gray correlation degree. The calculation formula is explained as follows,

$$\gamma 0\mathrm{i} = \frac{1}{m}\sum_{k}^{m}\gamma 0i(k) \qquad \text{(S27)}$$

S1.5. Industrial standards

This study aims to meet the requirements of seamless bonding in textile industries by setting target values to achieve valuable and key properties. The industry specifications for seamless bonding are shown in Table S6. The softening temperature of the hot melt adhesive should be between 70-100°C. High softening temperature caused many problems such as substrate shrinkage and color differences during the bonding process, and low bonding temperature could not be reached at softening temperature, ultimately problem in sufficient adhesion. The peel strength and shear strength represent the adhesion and cohesion between the adhesive and the nylon fabric, respectively. Therefore, optimized and standard values avoid problems such as delamination and slipping caused by insufficient adhesion or cohesion after bonding.

Table S6. objectives of this study to get the target

Testing items	Target values	Standard
softening point	70-100°C	ASTM D36
Peel strength	0.39 kg/cm above	ASTM D3330
shear strength	13 kg/cm^2 above	CNS 8306 L3141

Table S7. Factor response table for shear strength of dual-cure polyurethane hot melt adhesive

	A (kg/cm^2)	B (kg/cm^2)	C (kg/cm^2)
Level 1	29.71	29.01	29.55
Level 2	29.84	29.67	29.97
Level 3	29.53	30.40	29.56
Max	29.84	30.40	29.97
Min	29.53	29.01	29.55
effect	0.31	1.40	0.42
Sort	3	1	2

Table S8. The ANOVA analysis table for shear strength of dual-curing polyurethane hot melt adhesive

Control factor	SS (kg/cm^2)	DOF	Var	F	Contribution
A	0.15	2	0.07	18.11	4.27%
B	2.92	2	1.46	362.33	85.49%
C	0.34	2	0.17	42.37	10.00%
Error	0.01	2	0.00		
Combined Error	0.01	2	0.00		
Total	3.42	8			

Table S9. Experimentation of shear strength for Dual-Cure Polyurethane Hot Melt Adhesive

Major significant factor	Test 1 (kg/cm^2)	Test 2 (kg/cm^2)	Test 3(kg/cm^2)	Mean value (kg/cm^2)	S/N ratio
A2B3C2	33.91	33.94	34.02	33.96	30.62

Table S10. ANOVA Analysis Table of Peel Strength for Dual-curing Polyurethane Hot Melt Adhesive

Control Factor	Sum of Squares	Degrees of Freedom	Variance	F Distribution	Contribution
A	299.83	2	149.91	55.78	73.84%
B	85.99	2	42.99	16.00	21.18%
C	14.87	2	7.44	2.77	3.66%
error	5.38	2	2.69	-	-
Combined Error	5.38	2	2.69	-	-
Total	406.06	8	-	-	-

Table S11. Confirmation Experiment for Dual-Cure Polyurethane Hot Melt Adhesive Peel Strength

Primary significant factor	Test1	Test2	Test3	mean value	S/N ratio
A3B3C3	1.63	1.62	1.62	1.62	4.21

Table S12. Difference Sequence for Peel Strength and Shear Strength

	Peel strength S/N ratio	Shear strength S/N ratio
X_0	0.00	0.00
X_1	1.00	0.93
X_2	0.79	0.29
X_3	0.46	0.14
X_4	0.69	0.63
X_5	0.44	0.47
X_6	0.27	0.03
X_7	0.13	1.00
X_8	0.07	0.67
X_9	0.00	0.00

Table S13. Confirmation experiment output table (peel strength) for dual-cure polyurethane hot melt adhesive

Significant controlling factors	Test1	Test2	Test3	mean value	S/N ratio
A3B3C2	1.67	1.69	1.68	1.68	4.51

Table S14. Confirmation experiment output table (shear strength) for dual-cure polyurethane hot melt adhesive

Significant controlling factors	Test1	Test2	Test3	mean value	S/N ratio
A3B3C2	35.62	34.52	34.67	34.94	30.86

Table S15. Comparison of properties between multi-quality experiment and commercial hot melt adhesive

Analysis Item	Target Value	Commercial Hot Melt Adhesive	Unoptimized Experiment	Multi-quality Optimization Experiment	Gain (%)
Peel strength	0.39 kg/cm above	0.73	1.57	1.69	7.64
Shear strength	13kg/cm^2 above	19.36	33.21	35.62	7.25

Table S16. Comparison of processing conditions between optimized experiment and commercial hot melt adhesive

Analysis Item	Commercial Hot Melt Adhesive	Multi-quality Optimization Experiment
Processing Temperature (°C)	150-170	100
Processing Time (s)	20-30	20
Hot Pressing Pressure (psi)	40-60	15
